# Bilateral blindness following a stabbing assault: A case report

**DOI:** 10.1016/j.ijscr.2022.107162

**Published:** 2022-05-04

**Authors:** Azeddine Lachkar, Drissia Benfadil, Fahd Elayoubi

**Affiliations:** ENT and head and neck surgery department, University hospital Center Mohammed VI, Faculty of medicine and Pharmacy, Mohammed First University, Oujda, Morocco

**Keywords:** Aggression, White weapon, Bilateral blindness, Case report

## Abstract

**Introduction:**

Stabbing is relatively frequent in Morocco and constitutes a major public health concern. Our observation is interesting by the particularity and the aggressiveness of the trauma leading to an irreversible bilateral blindness.

**Case presentation:**

The present observation relates to a patient who was victim of a stabbing assault. The examination objectified a facial wound in front of the right lateral canthus with a broken weapon in place. An urgent craniofacial CT scan was performed showing a hyperdense penetrating path of the stabbing from the lateral wall of the right orbit, associated with a bursting of the globe, obliquely crossing the anterior ethmoid cells and severing the optic nerve on the left side. Surgical exploration was made with an extraction of the remaining part of the knife. Bilateral blindness was irreversible and the patient had a psychiatric follow up.

**Conclusion:**

Aggressions are a sad reality, expressing anger and violence on the part of a young adult, present in any society with often dreaded consequences.

## Highlights

• Stabbing is relatively frequent in Morocco and constitutes a major public health concern
• The clinical examination and CT scan of a facial trauma victim is essential, but should not delay treatment
• The evolution of the facial wound can be punctuated with complications and/or sequelae
• Prevention of facial wounds involves the promotion of education in addition to laws against aggression and aggressors.

## 1 Introduction

Any facial wound may at times be life-threatening, and can lead to significant functional, and aesthetic sequelae. The psychological and socio- professional repercussions must always be taken into consideration. Knife attacks are relatively frequent in Morocco, and constitute a major public health concern. The present observation is interesting because it concerns a patient who was victim of a stabbing assault causing irreversible bilateral blindness.

This work has been reported in line with the SCARE criteria [1].

## 2 Case presentation

**Fig. 1:**
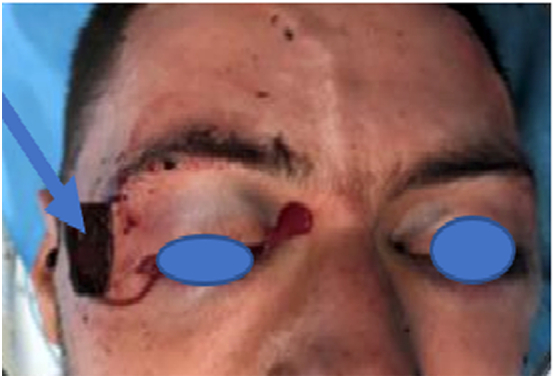
Patient upon arrival at the emergency room.

This is a 37-year-old patient with no significant pathological history who was admitted to the emergency room for the management of a serious facial wound following a stabbing attack. Clinical examination found a conscious patient with a Glasgow score of 15, hemodynamically, and respiratory stable. Exofacial examination revealed a facial wound over the right lateral canthus with the weapon in place without an exit port (Fig. 1).

**Fig. 2:**
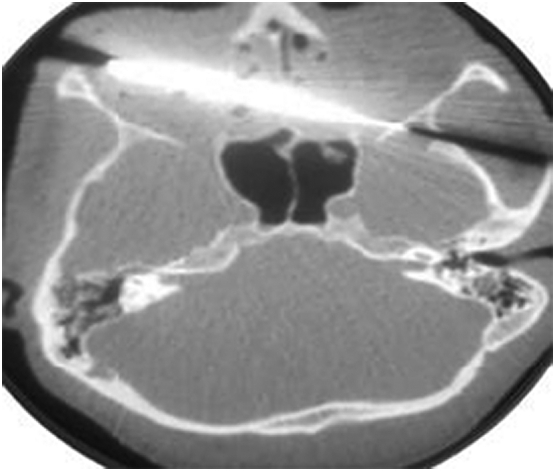
Axial CT-scan showing the knife shattering the right eyeball and severing the left optic nerve.

The ophthalmologic examination found negative light perception on both sides with a burst of the right eyeball. The rhinological examination revealed a moderate anterior epistaxis, for which anterior wicking was carried out by merocel packing. The neurologic exam was normal, and the remainder of the physical exam was unremarkable. A craniofacial computed tomography was performed urgently showing a hyperdense path of the knife starting from the lateral wall of the right orbit, with bursting of the globe, obliquely crossing the anterior ethmoid cells and severing the optic nerve on the left side. The parenchymal scan revealed a thin section of the right temporal extradural hematoma (Fig. 2).

The patient benefited from multidisciplinary management involving several teams: otolaryngologist, ophthalmologist, and neurosurgeon, the main operator surgeon is an ENT assistant professor.

Surgical exploration was performed via the right and left trans-conjunctival route in pre- extraction, per-extraction, and post-extraction.

**Fig. 3:**
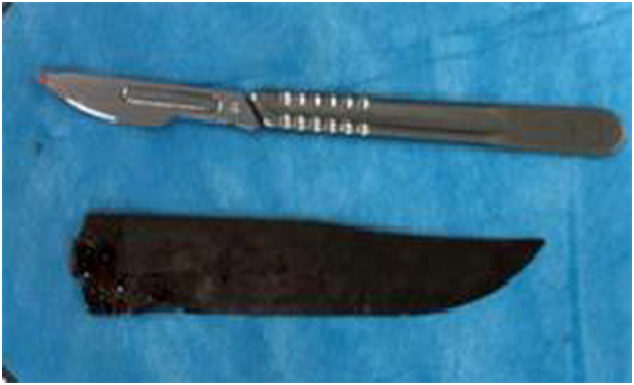
Stabbing after extraction.

The extraction was carefully and gradually performed with a 15 cm metal blade (Fig. 3).

The left optic nerve was entirely severed. The follow-up scan did not show any aggravation of the extradural hematoma, leading to a simple monitoring with an attitude of abstaining from neurosurgical intervention. The patient was put on antibiotic therapy of the amoxicillin/clavulanic acid type at a rate of three grams per day, anti-tetanus vaccination, and analgesic with simple operative consequences. No functional improvement was observed with a persistent absence of light perception bilaterally. A psychiatric follow up was prescribed for our patient.

## 3 Discussion

The face contains noble organs as well as almost all sensory organs, which are likely to be affected during trauma. Facial wounds remain the prerogative of young adults, with a clear predominance of men, this is largely explained by risky drunk driving, brawls and assaults [2]. Unilateral post-traumatic blindness is not uncommon, while bilateral blindness is exceptional.

Many cases of bilateral blindness by indirect trauma have been reported in the literature, Azonobi IR reported the case of a young male adult who suffered transient blindness following an assault in which he sustained deep cuts to the temporal and parietal regions of the scalp [3]. El Gatit A et al. describes a case of cortical blindness that followed successful surgical repair of two stab wounds in the heart in a 29-year old man, the patient recovered his vision three days later [4].

In our case, the blade of the knife penetrated between the outer orbital rim and the eyeball causing its bursting seen the width of the knife blade. The papery blade and the ethmoidal cells made of the papery bone easily constitute an area of weakness crossed by the blade of the knife which has reached the large wing of the left side severing the optic nerve.

The initial management is essential to prevent and limit as much as possible sequelae. Thus, the strengthening of emergency services by multidisciplinary teams considerably improves the care [5]. The latter begins in the emergency department, the interrogation and the clinical examination are essential to appreciate the state of consciousness, the hemodynamic state, and the airway freedom. The practitioner will be able to judge whether it is an isolated maxillofacial lesion or associated with a functional emergency as in our case and/or a vital emergency justiciable of a transfer to an Intensive Care Unit. The reference examination in the event of facial trauma is a CT scan, the completion of which should in no case delay treatment [6].

The extraction of the knife has to be avoided, otherwise this gesture can worsen the lesions, or lead to a potential hemorrhage, and can only be done in the operating room by allowing the control of the blade of the knife on all its route [5]. Removal of the knife can be performed in the interventional neuroradiology room after prior endovascular treatment to avoid potential hemorrhage [7]. Removal of a superficial cutting agent could be done easily but the deeper ones are more difficult to extract and are associated with more complications, especially, those in head and neck area. Hatem H. Al-Ahmady reported a case of a limited mouth opening in a child two months after a traumatic collision with a glass door. A part of the glass was impacted as a foreign body and has been misdiagnosed, its base was at the submandibular region touching hyoid bone and its apex was at the infratemporal region. The removal of the foreign body was done after gaining access across the old scar through a submandibular incision [8].

The evolution of facial wounds is most often favorable, especially when appropriate care has been instituted in time. However, this evolution can be punctuated with complications and/or sequelae especially if the lesion interests a noble element as in our case.

## 4 Conclusion

Facial attacks frequently affect young adults, affecting aesthetic or even functional prognosis of these patients, which can significantly impact their future life. Improving the management of facial wounds undoubtedly involves strengthening reception capacities, and the technical platform for emergencies [9].

Prevention, on the other hand, involves the promotion of education, public awareness, effective regulation of security, in addition to laws against aggression and aggressors.

## Provenance and peer review

Not commissioned, externally peer-reviewed.

## Funding

Have no funding to report.

## Ethical approval

The study committee of the university hospital center approves the favorable opinion to publish this work.

## Consent

Written informed consent was obtained from the patient for publication of this case report and accompanying images. A copy of the written consent is available for review by the Editor-in-Chief of this journal on request.

## Registration of research studies

Not applicable.

## Guarantor

Azeddine Lachkar.

## Availability of data and material

The datasets in this article are available in the repository of the ENT database, Chu Mohamed VI Oujda, upon request, from the corresponding author.

## CRediT authorship contribution statement

Azeddine Lachkar: the main operator surgeon (formulated the treatment plan).

Drissia Benfadil: analysed and performed the literature research.

Fahd Elayoubi: performed the examination and the scientific validation of the manuscript.

Azeddine Lachkar was the major contributors to the writing of the manuscript. All authors read and approved the manuscript.

## Declaration of competing interest

The authors have no conflicts of interest to declare.
